# 特异性抗体免疫组化法检测*EGFR*突变价值的*meta*分析

**DOI:** 10.3779/j.issn.1009-3419.2014.06.03

**Published:** 2014-06-20

**Authors:** 晴 马, 竞 王, 殿胜 钟, 超 宁, 畅 刘, 平 肖

**Affiliations:** 1 300052 天津，天津医科大学总医院肿瘤科 Department of Medical Oncology, Tianjin Medical University General Hospital, Tianjin 300052, China; 2 300052 天津，天津医科大学总医院，天津市肺癌研究所 Tianjin Lung Cancer Institute, Tianjin Medical University General Hospital, Tianjin 300052, China

**Keywords:** 免疫组化, DNA测序, 表皮生长因子受体, *Meta*分析, Immunohistochemistry, DNA sequencing, Epidermal growth factor receptor, *Meta* analysis

## Abstract

**背景与目的:**

已有的研究表明：表皮生长因子受体（epidermal growth factor receptor, *EGFR*）基因突变是非小细胞肺癌（non-small cell lung cancer, NSCLC）患者应用表皮生长因子受体酪氨酸激酶抑制剂（EGFR tyrosine kinase inhibitor, EGFR-TKI）治疗疗效的最重要的预测因子。*EGFR*基因突变的患者对于使用TKIs分子靶向药物治疗疗效更敏感。其突变检测对肺癌一线靶向治疗选择尤为关键。研究分析特异性抗体免疫组化法（immunohistochemistry, IHC）检测*EGFR*突变与DNA测序法比较的敏感度与特异度，明确该方法准确性及临床应用价值。

**方法:**

通过Pubmed数据库检索所有符合检索条件的文献，末次检索日期2013年3月26日，根据纳入和排除标准进行进一步筛选，采用诊断试验*meta*分析方法，分析特异性抗体免疫组化方法与DNA直接测序法对比的敏感度与特异度。

**结果:**

10篇文献纳入*meta*分析，L858R 1, 679例，E746-A750del 1, 041例，诊断比值比（diagnositic odds ratio, DOR）分别为225.17（95%CI: 55.67-910.69）和267.16（95%CI: 132.45-538.88）；SROC曲线AUC分别为0.948, 4（SEAUC=0.014, 4）和0.981, 3（SEAUC=0.009, 9），Q^*^统计量分别为0.888, 3（SEQ^*^=0.019, 2）和0.9397（SEQ^*^=0.019, 1）。

**结论:**

以上两种特异性抗体IHC鉴别*EGFR*突变的特异度高，灵敏度较高，作为筛查突变方法可行性高，具有一定的临床应用价值。

## Abstract (en)

**Background and objective:**

It has been proven that epidermal growth factor receptor (*EGFR*) mutation is the most important predictive factor for determining the effect of EGFR tyrosine kinase inhibitors (TKIs) applied to non-small cell lung cancer (NSCLC) patients. The patients with *EGFR* mutations response better to TKIs. To detect *EGFR* mutation has been particularly essential to select first-line treatment for lung cancer patients. To research and analyze the sensitivity and specificity of immunohistochemistry (IHC) using mutation specific antibodies in detecting *EGFR* mutations compared with DNA sequencing, and further evaluate the accuracy and clinical application value of IHC.

**Methods:**

All required articles in Pubmed database were searched. The deadline of retrieval was March 26, 2013. Then further screening the articles based on the inclusion and exclusion criteria. *Meta* analysis of diagnostic test was applied to analyze the sensitivity and specificity of IHC compared with DNA sequencing for the detection of *EGFR* mutations.

**Results:**

Ten articles were included in the *meta* analysis, there were 1, 679 samples in L858R group and 1, 041 samples in E746-A750del group. The DOR were 225.17 (95%CI: 55.67-910.69) and 267.16 (95%CI: 132.45-538.88) respectively; the AUC of SROC were 0.948, 4 (SEAUC=0.014, 4) and 0.981, 3 (SEAUC=0.009, 9) respectively; the *Q* values were 0.888, 3 (SEQ^*^=0.019, 2) and 0.939, 7 (SEQ^*^=0.019, 1) respectively.

**Conclusion:**

The specificity and sensitivity of IHC method using these two mutation-specific antibodies were relatively high. As a screening method for *EGFR* mutations, the IHC with mutation specific antibodies is of clinical value.

表皮生长因子受体（epidermal growth factor receptor, EGFR）是一种跨膜受体酪氨酸激酶，属于HER家族的一员。研究^[1]^发现，EGFR在50%-90%的非小细胞肺癌（non-small cell lung cancer, NSCLC）患者中高表达，参与肿瘤的血管新生、迁移和粘附过程，其扩增和突变已被认为是肺部肿瘤发生的主要机制之一。研究^[2-4]^表明，*EGFR*基因突变状态是决定EGFR酪氨酸激酶抑制剂（tyrosine kinase inhibitor, TKI）疗效最重要的预测因子。目前，已经报道的*EGFR*基因突变类型大约有60种^[1]^，包括外显子19的缺失、外显子18和21的单核苷酸的替换突变及20外显子的复制突变，其中外显子21的L858R和外显子19的缺失突变占突变的绝大多数，可达89%以上。

美国国立综合癌症网络（National Comprehensive Cancer Network, NCCN）NSCLC指南中明确指出，对于Ⅳ期非鳞NSCLC患者，应先行*EGFR*基因突变检测，如果存在*EGFR*基因突变，治疗上优先推荐EGFR-TKI。

目前*EGFR*突变检测方法较多，现有临床应用的方法中，直接测序法和ARMS法应用较广。DNA直接测序法，作为*EGFR*突变检测的金标准，可以检测所有的突变分析的区域，但其灵敏度较低，样本要求高，只能对含量大于30%的突变基因进行检测。ARMS法敏感，流程速度快、简单，数据分析要求低，但仅能检测已知突变，且试剂费用昂贵，临床中推广有一定困难。2009年Yu等^[5]^首次制备出了2种最常见的*EGFR*突变的特异性单克隆抗体——抗E746-A750缺失突变抗体和抗L858R点突变抗体，并应用于福尔马林固定、石蜡包埋组织的免疫组织化学（immunohistochemistry, IHC）检测。IHC作为常规病理检查手段，具有标本处理方法简单、快速，价格便宜，且可以在临床病理科进行。近年来多项临床独立研究^[6-15]^应用特异性抗体检测NSCLC患者*EGFR*突变检测与直接测序法比较，其敏感度44%-100%，特异度85%-99%。本文通过*meta*分析判定IHC法诊断准确度及临床应用价值。

## 1 材料与方法

### 1.1 检索策略

计算机检索Pubmed、中国医院知识仓库医学专题全文数据库（CNKI）、中国生物医学文献数据库（CBM disc）和万方数据库。检索时间：2009年1月-2013年2月。收集国内外公开发表的“关于特异性抗体免疫组化法检测*EGFR*突变价值”的文章。中文检索词为表皮生长因子受体突变、L858R点突变、抗体、E746-A750缺失突变、免疫组织化学法、非小细胞肺癌。英文检索词：epidermal growth factor receptor、EGFR、non-small cell lung cancer、NSCLC、E746-A750 deletion mutantion、immunohistochemical method、L858R point mutation。

### 1.2 纳入与排除标准

研究类型：使用*EGFR*突变特异性抗体L858R及E746-750del检测外显子21及外显子19突变情况，同时应用检测金标准DNA直接测序法比较其敏感度特异度的文献纳入标准：①研究类型为含有*EGFR*突变特异性抗体L858R及E746-A750del检测对NSCLC患者*EGFR*突变检测价值的前瞻性或回顾性研究；②研究对象采用DNA直接测序为金标准，文献需明确说明受试者病理类型；③文章提供了特异性抗体免疫组化检测在各病例组的真阳性（true positive, TP）、真阴性（true negative, TN）、假阳性（false positive, FP）、假阴性（false negative, FN）例数或通过文章提供的数据可以计算；④每组病例数均 > 20；⑤文献中*EGFR*突变检测采用统一可评价的IHC方法及标准（IHC阳性定义：10%以上肿瘤细胞胞膜染色定义为阳性，DNA直接测序标本均来源于NSCLC患者的FFPE标本）。

排除标准：①IHC与其他检测方法（如ARMS法）比较，而无直接测序法对照的文献，主要因为ARMS法虽为常用临床检查方法，敏感度特异度高但仅能检测已知突变，对于IHC法阳性预测值（positive predictive value, PPV）和阴性预测值（negative predictive value, NPV）统计有一定影响；②采用除以上两种特异性抗体进行免疫组化的检测；③EGFR免疫组化无统一判定标准的文献；④重复性实验中，发表较早或样本量较小的文献排除。

### 1.3 数据提取

所有纳入研究均提取以下内容：①研究人群基本情况；②各个研究的对于特异性抗体免疫组化法检测*EGFR*突变筛检试验的真阳性、真阴性、假阳性和假阴性，由2名作者按照上述标准独立纳入文献和提取资料，而后交叉核对，意见不一致时通过讨论解决。

### 1.4 数据处理和统计学分析

整理原始文献并摘录数据，由2名作者独立输入数据，用*meta*-Disc 1.4进行分析。用各研究精确估计量在受试者工作特征（receiver operator characteristic curve, ROC）曲线平面所形成的图像是否呈典型“肩臂”状分布进行各研究间由阈值效应引起的异质性分析；用*q*检验（inverse variance chi-squared test）进行异质性检验，如果同质性好（*P*≥0.05, *I*^2^≤25%），采用固定效应模型进行数据合并；若存在异质性（*P* < 0.05, *I*^2^ > 25%）采用随机效应模型分析。对比特异性抗体免疫组化方法与DNA直接测序法比较的敏感度、特异度，评判该方法的准确性。对各研究的原始数据（真阳性、假阳性、真阴性及假阴性的例数）进行整合，分别计算L858R及E746-A750del特异性抗体免疫组化的平均敏感度、特异度、比值比及各自的95%可信区间（confidence interval, CI）。采用Mose’s constant线性模型拟合SROC曲线，以诊断比值比（diagnositic odds ratio, DOR）、曲线下面积（aera under curve, AUC）和*Q*统计量评价免疫组化法对NSCLC患者*EGFR*突变诊断的准确度。以纳入*meta*分析的各研究的敏感度为Y轴，以（1-特异度）为X轴绘制SROC曲线，直观上评估诊断试验的准确性，曲线越靠近左上角，曲线下面积越大，其诊断准确性越高。按照*α*=0.05的检验标准进行统计学判断。

## 2 结果

### 2.1 检索结果及纳入研究文献

通过设定的检索词进行初步检索，共找到88篇文献。阅读文题和摘要排除62篇，初步纳入文献26篇。进一步阅读全文，排除未达到纳入标准的文献12篇，重复文献2篇，无法获得所需全部原始数据的文献2篇，最终纳入文献共10篇，如图 1所示，其中2篇仅涉及L858R抗体，未涉及E746-A750del抗体的免疫组化。

**1:**
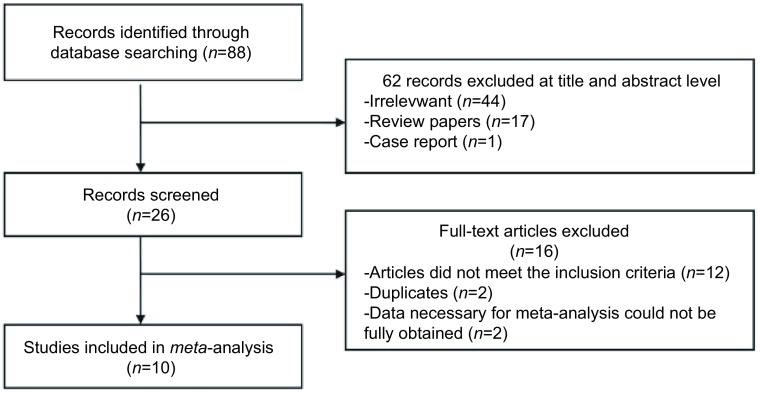
文献筛选流程图 Flow chart for study selection

### 2.2 纳入研究的基本特征

本文共纳入10项研究，E746-A750del免疫组化累计病例1, 679例，L858R免疫组化累计病例1, 041例，各研究免疫组化法例数、敏感度及特异度参见表 1。

**1:** 纳入文献的基本资料及免疫组化方法的相关数据 General parameters of included studies and the data of IHC

Included studies	Country	Experimental methods	Age(yrs)	*n*	L858R							E746-A750del					
			[Median(range)]		TP	TN	FP	FN	Sensitivity	Specificity		TP	TN	FP	FN	Sensitivity	Specificity
Brevet 2010^[6]^	America	IHC, DNA sequencing	---	194	20	171	2	1	95	99		23	161	2	8	74	99
Kato 2010^[7]^	America	IHC, DNA sequencing	59.9（27-88）	70	9	7	0	2	82	100		9	56	2	3	75	97
Kitamura 2010^[8]^	Japan	IHC, DNA sequencing	---	60	--	--	--	--^*^	79	100		--	--	--	--	83	100
Yu 2009^[5]^	China	IHC, DNA sequencing	---	340	24	193	2	2	88	100		23	196	0	3	88	100
Wu 2011 ^[9]^	China	IHC, DNA sequencing	65.2（27.2-86.9）	143	38	77	23	5	88	77		29	9	1	2	94	90
Angulo 2012^[10]^	Spain	IHC, DNA sequencing	60.1±8.9	136	--	--	--	--	89	100		--	--	--	--	100	100
Simonetti 2010^[11]^	Spain	IHC, DNA sequencing	64（36-85）	78	--	--	--	--	69	100		--	--	--	--	92	100
Nakamura 2010^[12]^	Japan	IHC, DNA sequencing	---	20	5	10	5	0	100	67		3	15	2	0	100	88
Hofman 2012^[13]^	France	IHC, DNA sequencing	---	61	--	--	--	--	90	99		--	--	--	--	--	--
Kozu 2011^[14]^	Japan	IHC, DNA sequencing	---	577	--	--	--	--	44	100		--	--	--	--	--	--
^*^Values for TP, TN, FP and FN that did offered by the references can be calculated by related data. TP: true positive; TN: true negative; FP: false positive; FN: false negative; IHC: immunohistochemistry.																	

### 2.3

纳入研究的方法学质量评价，结果见表 2。

**2:** 纳入研究的方法学质量评价 Methodological quality assessment of included studies

Included studies	1	2	3	4	5	6	7	8	9	10	11	12	13	14
Brevet 2010^[6]^	Unclear	Yes	Yes	Yes	Yes	No	Yes	Yes	Yes	Yes	Yes	Unclear	No	No
Kato 2010^[7]^	Yes	Yes	Yes	Yes	No	Yes	Yes	Yes	Yes	Yes	Yes	Yes	No	Yes
Kitamura 2010^[8]^	Yes	Yes	Yes	No	Yes	Yes	Yes	Yes	Yes	Yes	Yes	Unclear	No	Yes
Yu 2009^[5]^	Yes	Yes	Yes	Yes	No	No	Yes	Yes	Yes	Yes	Yes	Yes	No	No
Wu 2011^[9]^	Yes	Yes	Yes	Yes	Yes	Yes	Yes	Yes	Yes	Yes	Yes	Yes	No	No
Angulo 2012^[10]^	Yes	Yes	Yes	Yes	No	Yes	Yes	Yes	No	Yes	Yes	Unclear	No	No
Simonetti 2010^[11]^	Yes	Yes	Yes	Yes	Yes	Yes	Yes	Yes	Yes	Yes	Yes	Unclear	No	No
Nakamura 2010^[12]^	Yes	Yes	Yes	Yes	Yes	Yes	Yes	Yes	Yes	Yes	Yes	Yes	No	No
Hofman 2012^[13]^	Unclear	Yes	Yes	Yes	No	Yes	Yes	Yes	Yes	Yes	Yes	Unclear	No	No
Kozu 2011^[14]^	Unclear	Yes	Yes	Yes	No	Yes	Yes	Yes	Yes	Yes	Yes	Unclear	No	No
1: Was the spectrum of patients representative of the patients who will receive the test in practice? 2: Were objectives pre-specified? 3: Is the reference standard likely to correctly classify the target condition? 4: Is the time period between reference standard and index test short enough to be reasonably sure that the target condition did not change between the two tests? 5: Did the whole sample or a random selection of the sample receive verification using a reference standard of diagnosis? 6: Did patients receive the same reference standard regardless of the index test result? 7: Was the reference standard independent of the index test (*i.e.* the index test did not form part of the reference standard)? 8: Was the experiment of index test clearly described and repeatable? 9: Was the experiment of reference standard clearly described and repeatable? 10: Were the index test results interpreted without knowledge of the results of the reference standard? 11: Were the reference standard results interpreted without knowledge of the results of the index test? 12: Were the same clinical data available when test results were interpreted as would be available when the test is used in practice? 13: Were uninterpretable/ intermediate test results reported? 14: Were withdrawals from the study explained?														

### 2.4 *Meta*分析结果

#### 2.4.1 异质性检验

以DOR作为效应量，分别分析L858R、E746-A750del免疫组化与直接测序的异质性，*Q*检验显示Cochran-*Q*分别为20.31和5.64，*P* < 0.05，*P* > 0.05，*I*^2^分别为65.5%和0%，L858R抗体免疫组化研究间存在异质性，故以下分析选用随机效应模型。E746-A750del免疫组化采用固定效应模型。

#### 2.4.2 *Meta*分析

随机效应模型*meta*分析结果显示：应用特异性抗体免疫组化方法的合并敏感度、特异度、阳性似然比（positive likelihood ratio, PLR）、阴性似然比（negative likelihood ratio, NLR）和DOR比分别如图 2-图 6所示。

**2:**
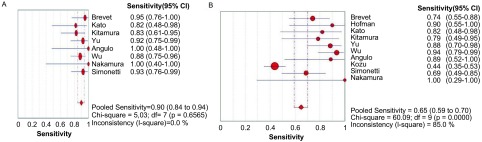
E746-A750del（A）和L858R（B）敏感度森林图 The forest plots of E746-A750del (A) and L858R (B) sensitivity

**3:**
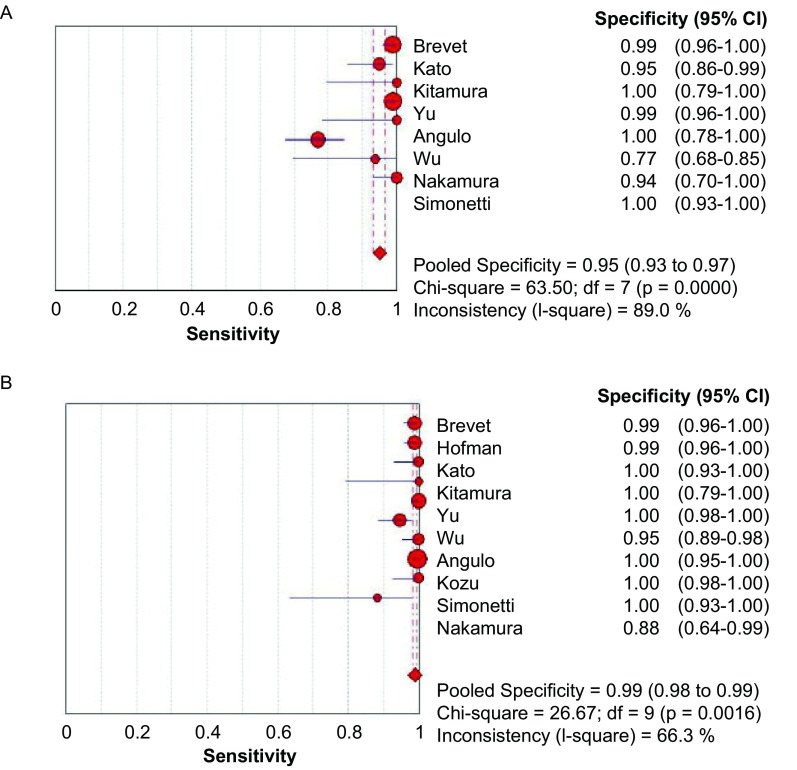
E746-A750del（A）和L858R（B）特异度森林图 The forest plot of E746-A750del (A) and L858R (B) specificity

**4:**
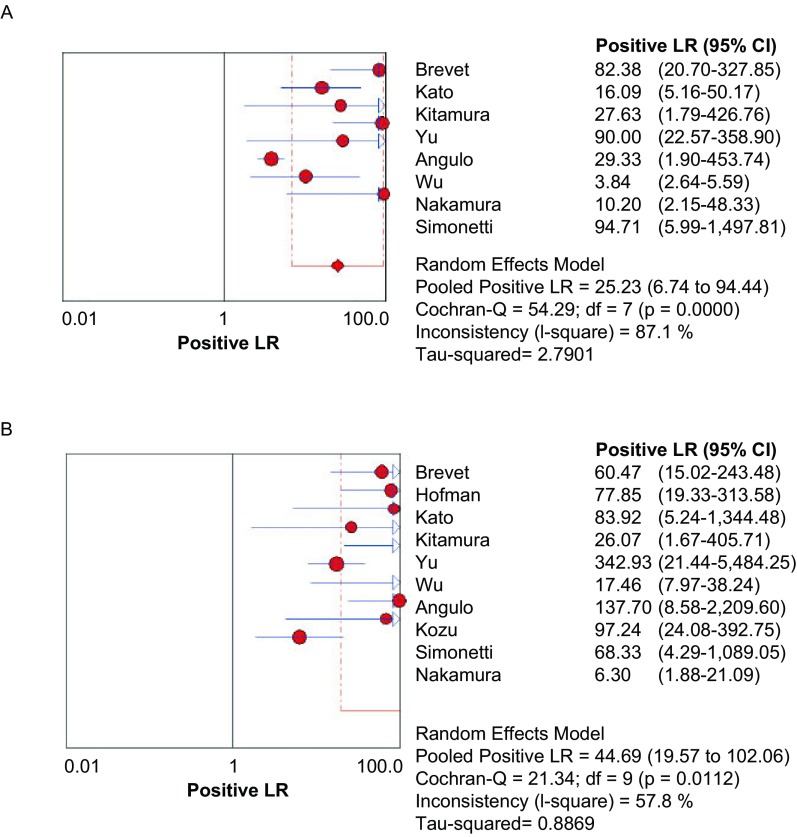
E746-A750del（A）和L858R（B）PLR森林图 The forest plot of E746-A750del (A) and L858R (B) PLR. PLR: positive likelihood ratio.

**5:**
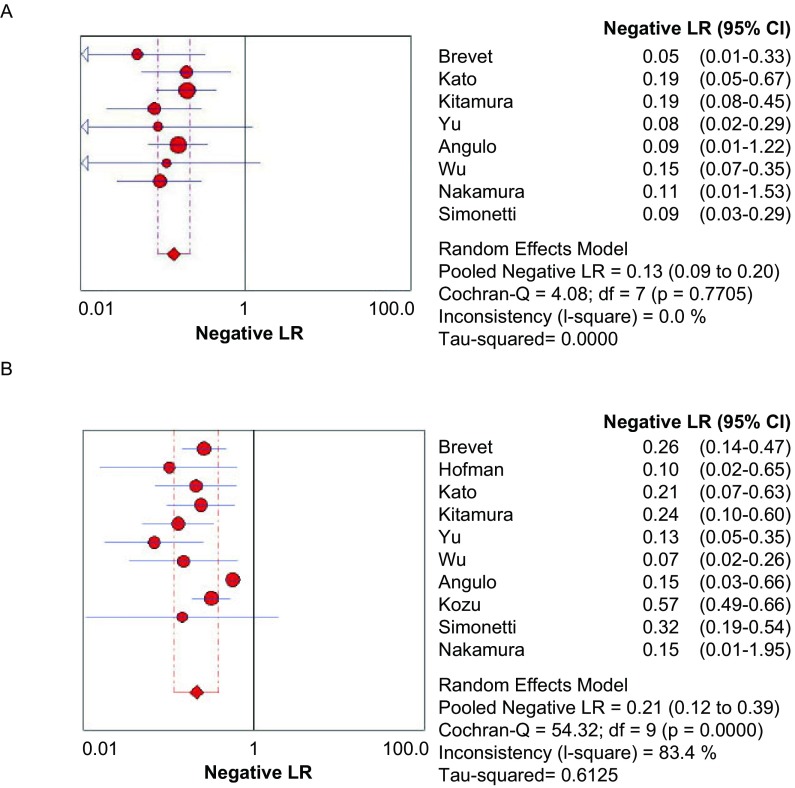
E746-A750del（A）和L858R（B）NLP森林图 The forest plot of E746-A750del (A) and L858R (B) NLP. NLR: negative likelihood ratio.

**6:**
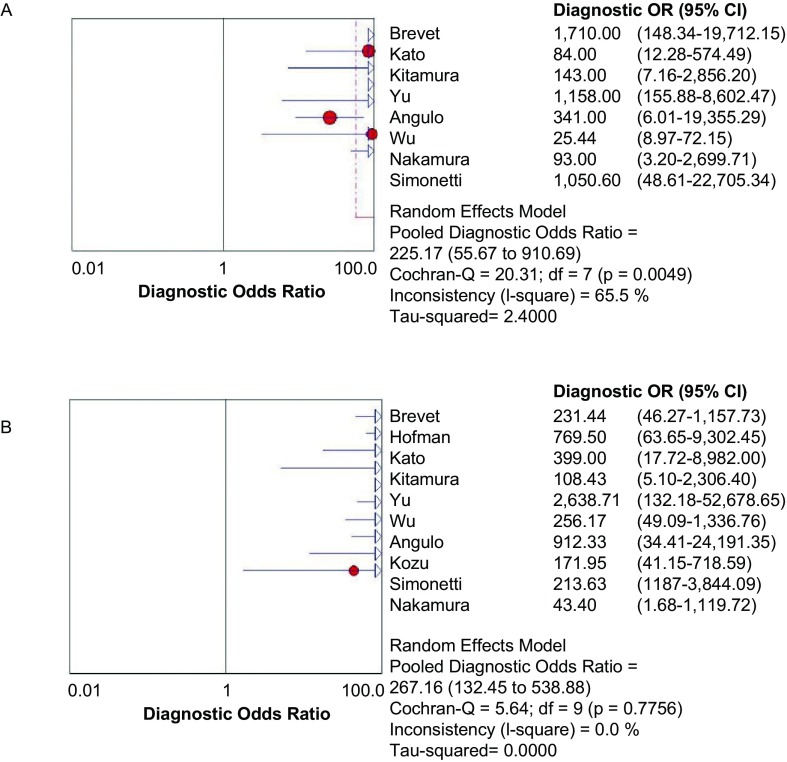
E746-A750del（A）和L858R（B）DOR森林图 The forest plot of E746-A750del (A) and L858R (B) DOR. DOR: diagnostic odds ratio.

图 2所示为E746-A750del和L858R对NSCLC诊断敏感度的森林图。E746-A750del鉴别NSCLC患者*EGFR*突变的平均敏感度为0.90（95%CI: 0.84-0.94, *P*=0.656, 5），L858R的平均敏感度为0.65（95%CI: 0.59-0.70, *P* < 0.001）。

图 3所示为E746-A750del和L858R对*EGFR*突变诊断特异度的森林图。E746-A750del鉴别*EGFR*突变的平均特异度为0.95（95%CI: 0.93-0.97, *P* < 0.001），L858R的平均特异度为0.99（95%CI: 0.98-0.99, *P*=0.001, 6）。

图 4所示为E746-A750del和L858R诊断*EGFR*突变的PLR分别为25.23（95%CI: 6.74-94.44, *P* < 0.001）和44.69（95%CI: 19.57-102.06, *P*=0.011, 2）。

图 5所示为E746-A750del和L858R诊断*EGFR*突变的NLR分别为0.13（95%CI: 0.09-0.20, *P*=0.770, 5）和0.21（95%CI: 0.12-0.39, *P* < 0.001）。

图 6所示为E746-A750del和L858R诊断*EGFR*突变的DOR分别为225.17（95%CI: 55.67-910.69, *P*=0.004, 9）和267.16（95%CI: 132.45-538.88, *P*=0.775, 6）。

#### 2.4.3 SROC曲线

由E746-A750del和L858R的SROC曲线，计算灵敏度对数与（1-特异度）对数的*Spearman*相关系数*ρ*，E746-A750del和L858R的*P*值分别为-0.500和0.382，*P*均 > 0.05，提示不存在阈值效应。SROC AUC两种检验方法分别为94.84%和98.13%，*Q*值为0.888, 3、0.939, 7（图 7）。将每个研究逐一排除后行敏感性分析，结果显示汇总灵敏度和特异度无明显改变，提示*meta*分析结果的稳定性较好。

**7:**
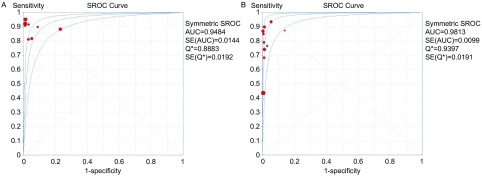
E746-A750del（A）和L858R（B）的SROC曲线 The SROC curve of E746-A750del (A) and L858R (B)

综上所述，E746-A750del和L858R特异性抗体免疫组化法鉴别*EGFR*突变，方法可靠，特异度高，灵敏度较高，IHC方法作为筛查突变方法可行性高，具有临床应用价值。

## 3 讨论

本文对纳入的10项研究进行*meta*分析，通过合并诊断效应量、拟合SROC曲线比较L858R、E746-A750del特异性抗体免疫组化与直接测序法比较对*EGFR*突变的诊断效能。结果显示E746-A750del鉴别NSCLC患者*EGFR*突变的平均敏感度为0.90（95%CI: 0.84-0.94），平均特异度为0.95（95%CI: 0.93-0.97）；L858R的平均敏感度为0.65（95%CI: 0.69-0.70），平均特异度为0.99（95%CI: 0.98-0.99）。两者结果综合显示特异性较高而敏感性稍差，结合相关文献，考虑敏感度差别主要源于该方法仅能检测已知最常见E746-A750del和L858R突变，而不能检测其他*EGFR*基因突变，如9 bp、12 bp、18 bp、21 bp和24 bp缺失或L861Q替代等。Dahabreh等^[15]^的一项*meta*分析报告显示，东亚人群中预测的特异性和敏感性分别为81%和81%。本研究结论与相关文献相符。

一般认为，PLR > 10或NLR < 0.1，基本可以确定或排除诊断。本研究得出的E746-A750del和L858R诊断*EGFR*突变的PLR分别为25.23（95%CI: 6.74-94.44）和44.69（95%CI: 19.57-102.6），提示两者阳性均可以辅助临床医师做出相应判断，具有临床应用价值。但E746-A750del和L858R的NLR分别为0.13（95%CI: 0.09-0.20）和0.21（95%CI: 0.12-0.39），提示二者阴性时不能排除*EGFR*突变的可能。

DOR反映诊断试验的结果与疾病的联系程度。取值> 1时，其值越大说明该诊断试验的判别效果较好；取值< 1时，正常人比患者更有可能被诊断试验判为阳性；取值=1时，表示该诊断试验无法判别正常人与患者。本研究中E746-A750del和L858R诊断*EGFR*突变的DOR分别为225.17（95%CI: 55.67-910.69）和267.16（95%CI: 132.45-538.88），提示诊断试验的判断效果好。

本文通过对可提供四格表数据的10篇文献，计算合并敏感度、特异度、PLR、NLR、DOR，行异质性分析后绘制SROC曲线，SROC曲线又名综合受试者工作特征曲线，不受异质性影响，可综合灵敏度与特异度信息，综合评价诊断试验的准确性，曲线以灵敏度为纵轴，以1-特异度为横轴，原理为通过TRP、FRP进行Logit变换将TRP与FRO间非线性关系转变为一种线性关系，利用最小乘法进行参数统计，建立SPOC曲线回归方程并获得评价诊断试验准确度的统计量。分析本文SROC曲线显示，L858R和E746-A750DEL的AUC分别为0.948, 4和0.981, 3，Q^*^统计量分别为0.888, 32和0.939, 7，曲线靠近左上角，曲线下面积大，说明以上两种特异性抗体IHC鉴别*EGFR*突变的准确度均较高。本文纳入的研究间存在异质性，经*Spearman*相关系数检验，异质性与阈值效应无关，仍需做进一步做*meta*回归，寻找异质性的可能来源。

本次*meta*分析的局限性：①*meta*分析的局限性：检索到的文献不够全面。检索范围局限在已经发表的研究，对于未公开发表的研究，如会议论文无法获取，可能漏检一些灰色文献；检索语种局限于中文和英文，可能会漏检其它语种的相关研究；②纳入研究的局限性：特异性抗体IHC作为诊断性试验，采用盲法检测和盲法判断可尽量减少诊断的倾向性，而多数研究未报告是否采用盲法检测，存在测量偏倚的可能性。

综上所述，目前的IHC可以检测EGFR最常见外显子19缺失和21外显子L858R点突变这两种突变，其灵敏度与特异性与直接测序法比较无明显差别，且简单易行，具有一定临床应用价值，有望成为NSCLC患者*EGFR*突变检测的常规程序。
